# Clinical Profile, Complications and Trends of Ocular Anaesthesia in a Multi-tier Ophthalmology Network in India: An Eight-Year Experience

**DOI:** 10.7759/cureus.57564

**Published:** 2024-04-03

**Authors:** Dilshad Kauser, Anthony Vipin Das, Gazella B Warjri, Koshy P George, Raja Narsing Rao, Sriramulu Pediredla

**Affiliations:** 1 Anaesthesiology, L V Prasad Eye Institute, Hyderabad, IND; 2 Ophthalmology, L V Prasad Eye Institute, Hyderabad, IND

**Keywords:** india, big data, emr, electronic medical records, ocular anaesthesia

## Abstract

Introduction: To describe the clinical profile, complications and trends of ocular anaesthesia in a multi-tier ophthalmology network in India.

Methods: This retrospective hospital-based study included 417,622 patients presenting between January 2013 and December 2020. Patients who were administered either topical, local or general anaesthesia for ocular surgery in at least one eye were included as cases. The data were collected using an electronic medical record system.

Results: Among the 417,622 patients, local anaesthesia was administered to 280,638, (67.2%) patients and was the most commonly administered type followed by topical anaesthesia in 84,117 (20.14%) patients. The most common complication encountered in administering local anaesthesia was retrobulbar haemorrhage in 103 (0.037%) patients followed by lid haematoma in 49 (0.017%) patients. Tooth damage occurred in 40 (0.076%) patients followed by delayed recovery in 30 (0.057%) patients during general anaesthesia. The trend of local anaesthesia decreased (83.48% vs 53.36%), whereas the trend of topical anaesthesia increased (8.61% vs 32.42%) over the study period.

Conclusion: There is a notable trend towards the adoption of less invasive anaesthetic methods, particularly in common surgeries such as cataract, intravitreal injection, and vitreoretinal surgery. However, despite this trend, a significant proportion of oculoplastic/orbital surgeries, trauma, and strabismus surgeries continue to be performed under general anaesthesia. These observations underscore the ongoing evolution of ocular anaesthesia practices, reflecting advancements in surgical techniques and patient preferences.

## Introduction

Vision impairment affects approximately 2.2 billion people worldwide, with half of these cases having preventable causes of impairment that can be managed or addressed [1]. A huge number of these cases would require some ophthalmic surgery. An enormous number of these cases would require some ophthalmic surgery, with all of them requiring some form of anaesthesia. Historically, general anaesthesia was the only method available for ocular surgeries. The first ever mention of topical anaesthesia was in 1884, when 4% cocaine was used [2], until the concept of local anaesthesia was described by Atkinson in 1936 [3]. Retrobulbar injections slowly moved to the peribulbar in view of the numerous complications being reported [4-6]. With the passage of time, ophthalmic procedures have shifted from in patient procedures to day care, as it becomes a more cost-effective, convenient affair for the patient. With more patients on anticoagulants and anti-platelets, a shifting trend toward topical anaesthesia makes it more favourable. There is still a paucity of literature on the experience of the administration of ocular anaesthesia for ocular surgery in the Indian population. The purpose of the study is to present the clinical profile, complications and trends of types of ocular anaesthesia in a multi-tier ophthalmology network in India using electronic medical record-driven analytics.

## Materials and methods

Study design, period, location and approval

This retrospective hospital-based study included all patients presenting between January 2013 and December 2020 to a multi-tier ophthalmology network located in India [7]. The patient, or the parents or guardians of the patient, filled out a standard consent form for electronic data privacy at the time of registration. None of the identifiable parameters of the patient were used for the analysis of the data. The clinical data of each patient who underwent a comprehensive ophthalmic examination was entered into a browser-based electronic medical records system (eyeSmart EMR) by uniformly trained ophthalmic personnel and supervised by an ophthalmologist using a standardised template [8]. The study adhered to the Declaration of Helsinki and was approved by the Institutional Ethics Committee.

Cases

A total of 417,622 patients underwent ocular surgery at the tertiary and secondary centres of the multi-tier ophthalmology network during the study period. The eyeSmart EMR was screened for patients who were administered either topical, local (peribulbar, sub-Tenon or retrobulbar block) or general anaesthesia for ocular surgery in at least one eye and were included as cases.

Data retrieval and processing

We retrieved the data of 417,622 patients from the electronic medical record database and segregated it into an Microsoft Excel sheet (Microsoft Corporation, Redmond, USA) for this study. The columns included data on patient demographics, surgery performed, ocular anaesthesia administered and complications information and were exported for analysis. The Excel sheet with the required data was then used for analysis using the appropriate statistical software.

Statistical analysis

Descriptive statistics using mean±standard deviation and median with interquartile range (IQR) were used to elucidate the demographic data. All tables for age, gender, surgery performed, ocular anaesthesia administered, and complications were drawn using Microsoft Excel (Microsoft Corporation, Redmond, USA). The chi-square test (StataCorp. 2015., Stata Statistical Software: Release 14; StataCorp LP, College Station, TX, USA) was used for the calculation of p-values.

## Results

**Figure 1:**
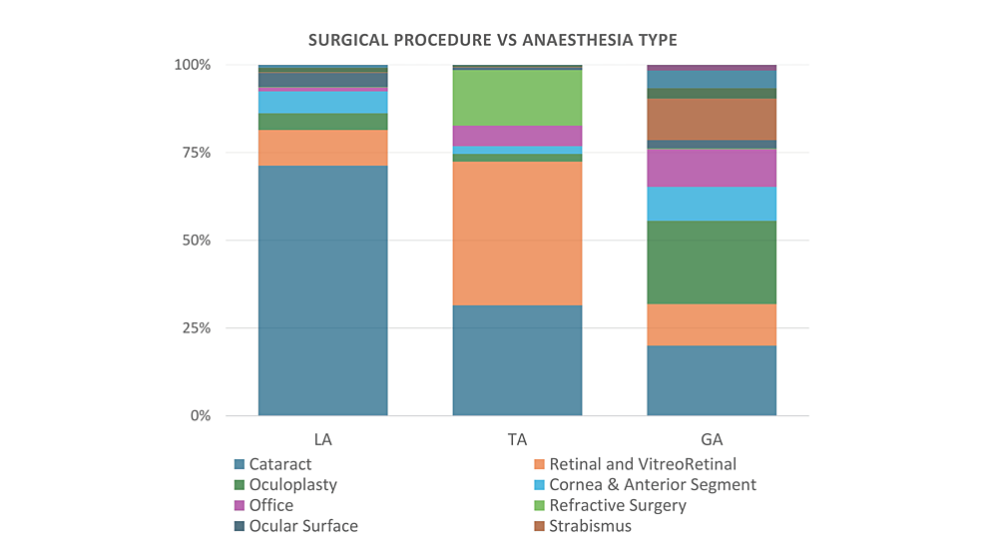
Graph showing the surgical procedure performed and the anaesthesia used: local anaesthesia (LA), topical anaesthesia (TA) and general anaesthesia (GA).

**Figure 2:**
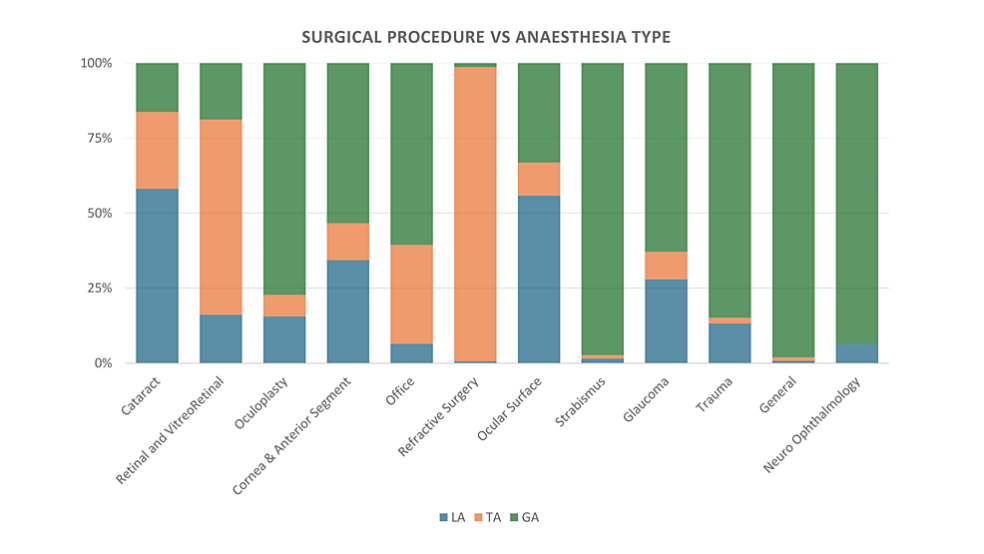
Graph showing the distribution of anaesthesia among the different surgical procedures. Office: Bandage contact lens placement, syringing and probing, corneal scraping and electrolysis. General: Examination under anaesthesia, corneal scraping. Neuro-ophthalmology: Optic nerve decompression and optic nerve sheath fenestration. LA: local anaesthesia, TA: topical anaesthesia, GA: general anaesthesia.

**Figure 3:**
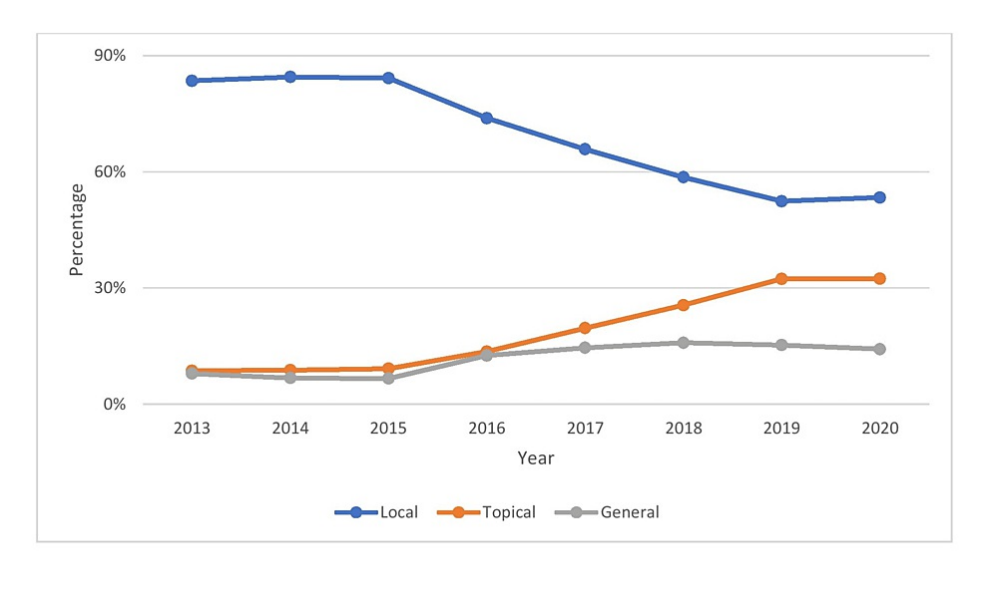
Yearly trends of the different methods of anaesthesia.

Overall, 417,622 patients were administered either topical, local, or general anaesthesia for ocular surgery in at least one eye during the study period. The overall summary comparing all three types of ocular anaesthesia is detailed in Table 1. Figure 1 shows the distribution of surgeries performed according to the anaesthesia method used, while Figure 2 shows the distribution of anaesthesia among the different surgical procedures. The yearly trends of the three types of ocular anaesthesia are detailed in Figure 3.

**Table 1:** Overview of topical, local and general anaesthesia. *Bandage contact lens placement, syringing and probing, corneal scraping and electrolysis. **Examination under anaesthesia, corneal scraping. #Optic nerve decompression and optic nerve sheath fenestration.

Gender	Topical	%	Local	%	General	%	Total
Male	49,574	22.11%	1,43,223	63.89%	31,383	14.00%	2,24,180
Female	34,543	17.86%	1,37,416	71.04%	21,483	11.11%	1,93,442
Age group							
0-10 yrs	2161	7.12%	106	0.35%	28,091	92.53%	30,358
11-20 yrs	2934	17.11%	4191	24.45%	10,019	58.44%	17,144
21-30 yrs	13,317	43.26%	12,668	41.15%	4799	15.59%	30,784
31-40 yrs	5825	22.61%	16,942	65.75%	3001	11.65%	25,768
41-50 yrs	9928	20.91%	35,119	73.97%	2432	5.12%	47,479
51-60 yrs	19,583	21.12%	70,832	76.39%	2310	2.49%	92,725
61-70 yrs	20,604	17.12%	98,122	81.55%	1592	1.32%	1,20,318
71-80 yrs	8398	18.36%	36,810	80.49%	526	1.15%	45,734
81-90 yrs	1305	18.65%	5601	80.04%	92	1.31%	6998
91-100 yrs	62	19.75%	248	78.98%	4	1.27%	314
Type of surgery							
Cataract	26,473	11.17%	2,00,066	84.38%	10,558	4.45%	2,37,097
Intravitreal injection	34,422	94.33%	1215	3.33%	854	2.34%	36,491
Vitreoretinal surgery	1852	5.67%	27,785	85.07%	3024	9.26%	32,661
Oculoplasty	1868	6.71%	13,439	48.24%	12,549	45.05%	27,856
Cornea and anterior segment	1863	7.65%	17,405	71.45%	5092	20.90%	24,360
Minor procedures*	4920	35.60%	3218	23.28%	5683	41.12%	13,821
Refractive surgery	13,303	96.98%	298	2.17%	116	0.85%	13,717
Ocular surface	664	5.05%	11,227	85.40%	1255	9.55%	13,146
Strabismus	119	1.73%	530	7.68%	6249	90.59%	6898
Glaucoma	372	6.48%	3770	65.68%	1598	27.84%	5740
Trauma	96	1.95%	2185	44.40%	2640	53.65%	4921
General**	17	1.90%	35	3.91%	843	94.19%	895
Neuro-ophthalmology#	0	0.00%	5	26.32%	14	73.68%	19

Local anaesthesia 

There were 280,639 (67.2%) patients who were administered local anaesthesia. There were 224,180 (53.68%) male and 193,442 (46.31%) female patients. Most patients were adults in 279,336 (99.54%) patients, followed by paediatric in 1,303 (0.46%) patients. The mean age of the patients was 57.7±13.89 years, while the median age was 61 (IQR: 51-67) years and the mode was also 61 years. The most common age group of the patients was distributed between 61 and 70 years (n = 98,122; 34.96%), followed by 51 and 60 years (n = 70,832; 25.24%). Cataract surgery accounted for the majority of surgical procedures requiring local anaesthesia in 200,066 (47.91%) patients. The most common complications encountered were retrobulbar haemorrhage in 103 (0.037%) patients, lid haematoma in 49 (0.017%) patients and globe perforation in 11 (0.004%) patients. The yearly trend of local anaesthesia decreased from 83.48% in 2013 to 53.36% in 2020, which was statistically significant (p<0.001).

General anaesthesia 

There were 52,866 (12.66%) patients who were administered general anaesthesia. There were 31,383 (59.36%) male and 21,483 (40.64%) female patients. The majority of the patients were paediatric 35,555 (67.25%) patients, followed by adults 17,311 (32.75%) patients. The mean age of the patients was 16.58±18.27 years while the median age was 9 (IQR: 3-23) years and mode was one year. The most common age group of the patients was distributed between 0 and 10 years (n = 28,091; 53.14%), followed by 11 and 20 years (n = 10,019; 18.95%). Oculoplasty surgery accounted for the majority of surgical procedures requiring general anaesthesia in 12,549 (3%) patients followed by cataract surgery in 10,558 (2.53%) patients. The most common complications encountered were tooth damage in 40 (0.076%) patients, followed by delayed recovery in 30 (0.057%) patients. A detailed list of general anaesthesia complications is listed in Table 2. The yearly trend of general anaesthesia increased from 7.91% in 2013 to 14.21% in 2020, which was statistically significant (p<0.001).

**Table 2:** Complications associated with general anaesthesia.

Complications	N	%
Tooth damage	40	0.076%
Delayed recovery	30	0.057%
Bronchospasm	26	0.049%
Apnea	21	0.040%
Laryngospasm	19	0.036%
Bradycardia	19	0.036%
Cardiac arrhythmia	9	0.017%
Hypotension	4	0.008%
Tube displacement	3	0.006%
Seizures	1	0.002%
Pulmonary edema	1	0.002%
No complications	52,693	99.673%
Grand total	52,866	100.000%

Topical anaesthesia 

There were 84,117 (20.14%) patients who were administered topical anaesthesia. There were 49,574 (58.94%) male and 34,543 (41.06%) female patients. Most patients were adults 80,685 (95.92%) patients, followed by paediatric 3,432 (4.08%) patients. The mean age of the patients was 50.18±19.09 years, while the median age was 55 (IQR: 35-65) years and the mode was 61 years. The most common age group of the patients was distributed between 61 and 70 years (n = 20,604; 24.49%), followed by 51 and 60 years (n = 19,583; 23.28%). Intravitreal injections accounted for the majority of surgical procedures requiring topical anaesthesia in 34,422 (40.92%) patients, followed by cataract surgery in 26,473 (31.47%) patients. The distribution of vitreoretinal procedures and intravitreal injections is given in Table 1. There were no significant complications documented with the use of topical anaesthesia. The yearly trend of topical anaesthesia increased from 8.61% in 2013 to 32.42% in 2020, which was statistically significant (p<0.001).

## Discussion

There was a trend of significant reduction of local anaesthesia with an increase in topical anaesthesia. The most common complications documented after local anaesthesia were retrobulbar haemorrhage, lid haematoma and globe perforation, with all complications at a rate of <0.01%, whereas there were no documented significant complications post-topical anaesthesia. The majority of cases under local anaesthesia were cataract surgeries, followed by vitreoretinal procedures. Oculoplasty surgeries were the dominant surgeries under general anaesthesia, and there was a statistically significant increase in the number of general anaesthesia surgeries from 2013 to 2020. The most common complications encountered during general anaesthesia were tooth damage in 0.076% of patients, followed by delayed recovery in 0.057% of patients.

Cataract surgery, done with the aim of visual rehabilitation, was the most common ophthalmic surgery performed worldwide but has mainly shifted to local anaesthesia, with an audit showing that 46.9% were under sub-Tenon’s anaesthesia, followed by 22.3%, which were under topical anaesthesia and peribulbar 19.5% [9]. Local complications that can occur due to local anaesthesia are retrobulbar haemorrhage [10], persistent ptosis (1.1%) [11] and mechanical needle trauma complications. Mechanical trauma complications would include vitreous haemorrhage, retinal detachments, retinal tears/lacerations, choroidal haemorrhage, and hypotony [12,13]. The risk of globe perforation increases with the presence of staphyloma in myopia [14]. Other complications include an increase in IOP leading to central retinal artery occlusion [13]. In our study, the main complications were retrobulbar haemorrhage, lid haematoma and globe perforation.

Vitreoretinal surgeries used to be done under general anaesthesia, but local anaesthesia has largely replaced it in the present day. About 85-90% of them are being done under local anaesthesia now [15]. In our study, 41.16% of vitreoretinal surgeries were performed under local anaesthesia and intraocular injections accounted for 49.78% of surgeries performed under topical anaesthesia. Improvements in vitreoretinal procedures, like the use of a smaller gauge for vitrectomy, have shifted the trend to local anaesthesia [16].

Penetrating ophthalmic injuries are almost an absolute indication for general anaesthesia to prevent the extrusion of the contents of the globe following local anaesthesia. However, repairs done under local anaesthesia have also been reported, where the wound length is <8 mm and the extension from the limbus is <4 mm [17]. In our cases, most penetrating ophthalmic injuries (53.65%) were operated under general anaesthesia, closely followed by local anaesthesia (44.4%). The rate of general anaesthesia was higher due to the higher proportion of the paediatric age group and the need for the exploration of larger wounds and to ensure the cooperation of patients.

The oculocardiac reflex has equal rates of occurrence in peribulbar and topical anaesthesia cases; therefore, one has to always be vigilant, even during procedures being performed under topical anaesthesia [18].

Topical anaesthesia is not always desirable for most ophthalmic surgeries as it does not provide akinesia, but it is popular for cataract surgery [19], although many surgeons prefer to add intracameral supplementation. The most common form of intracameral supplementation is lidocaine in its preservative-free form [20]. The safety in regard to corneal endothelium has been proven [21,22]. The most common agents used for topical anaesthesia are proparacaine, tetracaine, oxybuprocaine, and lidocaine [23]. In our patients, we have used proparacaine as per the Drug Formulary approved by the Drug Committee of the Organisation. Topical anaesthesia rates in cataract surgery have increased over the years due to the improvement of cataract surgery procedures like phacoemulsification and the faster recovery time.

There is an increasing trend in the adoption of topical anaesthesia in ocular surgery, although some surgeries would still require local anaesthesia. These include open intraocular surgeries like penetrating keratoplasty to prevent an increase in vitreous pressure if akinesia is not achieved fully and during enucleation or evisceration if general anaesthesia is not possible.

General anaesthesia, although a more costly affair, is indicated in children, if the procedure is time-consuming or painful, or when local anaesthesia is not possible or due to patient preference [24]. In our study, general anaesthesia was performed more in oculoplastic surgeries, which include external and endoscopic dacryocystorhinostomies, which shows a similar trend as other studies [25,26].

Our study is the largest series in literature for ocular anaesthesia methods. However, being a retrospective study, the limitations associated with a retrospective study cannot be overlooked. We could not assess the risk factors associated with the complications seen in the various modes of anaesthesia. The patient's preferences and the influence of regional practices could also not be assessed. Prospective studies are warranted for these.

## Conclusions

In conclusion, our retrospective analysis provides a comprehensive overview of ocular anaesthesia practices, complications, and trends within a multi-tier ophthalmology network in India. Our findings indicate a notable trend toward the adoption of less invasive anaesthetic methods, particularly in common surgeries such as cataract, intravitreal injection, and vitreoretinal surgery. However, despite this trend, a significant proportion of oculoplastic/orbital surgeries, trauma, and strabismus surgeries continue to be performed under general anaesthesia. These observations underscore the ongoing evolution of ocular anaesthesia practices, reflecting advancements in surgical techniques and patient preferences. Further research is warranted to optimise anaesthesia choices and enhance patient outcomes across diverse ophthalmic procedures. 
